# Isolated scaphoidectomy for type II SLAC and SNAC wrists: retrospective case-series at long-term follow-up

**DOI:** 10.1007/s00402-026-06350-z

**Published:** 2026-05-24

**Authors:** Maurizio Altissimi, Elisabetta Pataia, Michela Saracco, Luca Braghiroli

**Affiliations:** 1 Hand Surgery Unit, Clinical Institute Porta Sole, Perugia, Italy; 2 University of Perugia, Perugia, Italy; 3 Department of Orthopaedics, University Hospital “A.Gemelli”, Rome, Italy; 4 Department of Public Health, Orthopedic and Hand Surgery, University of Naples “Federico II”, Naples, Italy; 5 Department of Hand Surgery and Microsurgery, “S. Maria” Hospital, Terni, Italy

**Keywords:** Isolated scaphoidectomy, SNAC, SLAC, Wrist osteoarthritis, Long-term follow-up

## Abstract

**Backgrounds:**

Stage II scapholunate advanced collapse (SLAC) and Scaphoid non-union advanced collapse (SNAC) wrists are currently treated either by scaphoidectomy and Four Corner Fusion or by Proximal Row Carpectomy. In our retrospective study, isolated scaphoidectomy demonstrated clinical non-inferiority when compared to standard procedures and could be supplemented by a Four Corner Fusion only at a later time if needed.

**Methods:**

Between 2006 and 2011, 13 patients / 14 wrists (mostly males, manual workers) underwent the surgical procedure. 8 patients were affected by SNAC and 5 by SLAC. The average age was 56,5. All patients were manual workers. The mean follow-up was 15 years. These patients were reviewed a first time in 2012 and once again in 2024. Subjective outcome, DASH score, return to work, ROM and grip strength were evaluated. Radiographic assessment was done in all 14 wrists at the first follow-up and in 6 at the last long-time review. Statistical analysis was conducted using the SPSS software.

**Results:**

12 patients had resumed the same work at a mean of 3 months after surgery. Only one patient required a Four Corner Fusion after 2 years. 11 of the 13 enrolled patients declared their satisfaction for the procedure at the longest follow-ups. Clinical results remained stable over time. Radiological deterioration was seen in 6/13 wrists at the first follow-up and in all the 6 patients that could be investigated with an X-ray at the second long term follow-up. Lunate-Capitate arthrosis and Lunate DISI pattern were the radiological features observed without carpal collapse.

**Conclusion:**

Isolated scaphoidectomy for type II SLAC and SNAC wrist is a reliable and simple surgical procedure which could be proposed only after careful patient selection. This procedure has maintained a high level of satisfaction at long-term follow-up despite the radiological deterioration. It is far less invasive and requires a shorter time of functional recovery than Four Corner Fusion and Proximal Row Carpectomy in elderly or low-demanding patients with good pain relief. In case of failure, isolated Scaphoidectomy can be supplemented by a Four Corner Fusion at a later time if needed.

**Supplementary Information:**

The online version contains supplementary material available at 10.1007/s00402-026-06350-z.

## Introduction

 Scapholunate ligament lesions and scaphoid non-union can lead to osteoarthritic degeneration of the radio-carpal joint. The progression of osteoarthritic changes in scapholunate advanced collapse (SLAC) wrists was originally described by Watson and Ballet in 1984 and classified in four stages [1]. Scaphoid non-union advanced collapse (SNAC) was described by Vender et al. in 1987 [2]. SLAC and SNAC are very similar clinical conditions since they both lead to a series of predictable degenerative changes. In SLAC and SNAC stage II wrist osteoarthritic changes are still limited to the radio-scaphoid joint while the midcarpal joint is still not involved [3]. Four Corner Fusion (4CF) or Proximal Row Carpectomy (PRC) are two recommended surgical options in stage II and both procedures are able to provide improvements in pain and wrist function [4–6]. The superiority of one procedure over another has yet to be established [7].

Although both procedures proved to be effective, the midcarpal joint arthrodesis (the 4CF) or the entire proximal row carpectomy are more invasive surgical procedures with long recovery time. Moreover, both procedures have possible complications. Non-union, hardware issues, dorsal impingement and prolonged postoperative immobilization are possible complications in case of 4CF [8–12]. When the PRC is performed, the radio-capitate joint becomes incongruent and this can lead to detrimental contact areas, shearing forces and subsequent early capitate wear with corresponding radiographic degenerative changes [13–15].

On the other hand, more conservative treatment for SLAC and SNAC type II wrists have been proposed by some authors. The distal scaphoid pole resection has been shown to give good clinical results in stage II SNAC wrists [16–18].

Scaphoidectomy and midcarpal tenodesis has also been published by Luchetti et al. and proposed for the treatment of stage II SLAC and SNAC wrists with good outcomes [19, 20].

Trumble et al. proposed scaphoidectomy and dorsal capsulodesis in stage II SLAC and SNAC wrists achieving good pain relief and improvement of grip strength at a medium term follow-up [21].

In line with Trumble et al., we proposed an unusual surgical procedure, isolated scaphoidectomy, compared to standard procedures, such as the excision of the scaphoid bone with midcarpal fusion.

This study presents the long-term outcomes of a series of 14 Isolated Scaphoidectomy for stage II SLAC and SNAC wrists in 13 patients.

## Materials and methods

We retrospectively studied a total of 13 patients (14 wrists), 2 women and 11 men, who underwent isolated scaphoidectomy for stage II SLAC and SNAC wrist osteoarthritis. The senior author (A.M.) performed all the surgical procedures by a volar approach, between 2006 and 2011.

The inclusion criteria were: adults (> 50 years) affected by symptomatic wrist stage II SNAC or SLAC with long-term documented follow-up after isolated scaphoidectomy.

The exclusion criteria were: previous surgical procedures on the carpal bones, patients suffering from inflammatory diseases (rheumatoid arthritis) and patients affected by stage III or IV wrist osteoarthritis.

The mean age of the enrolled patients at the time of surgery was 56,5 years (min: 50; max: 65; SD: 4,86). The right side was involved in 9 cases. Eight patients were affected by SNAC disease, 5 patients by SLAC. The mean duration of symptoms before surgery was 3,76 years (min: 1; max: 6; SD: 2,40).

### Surgical procedure

**Fig. 1:**
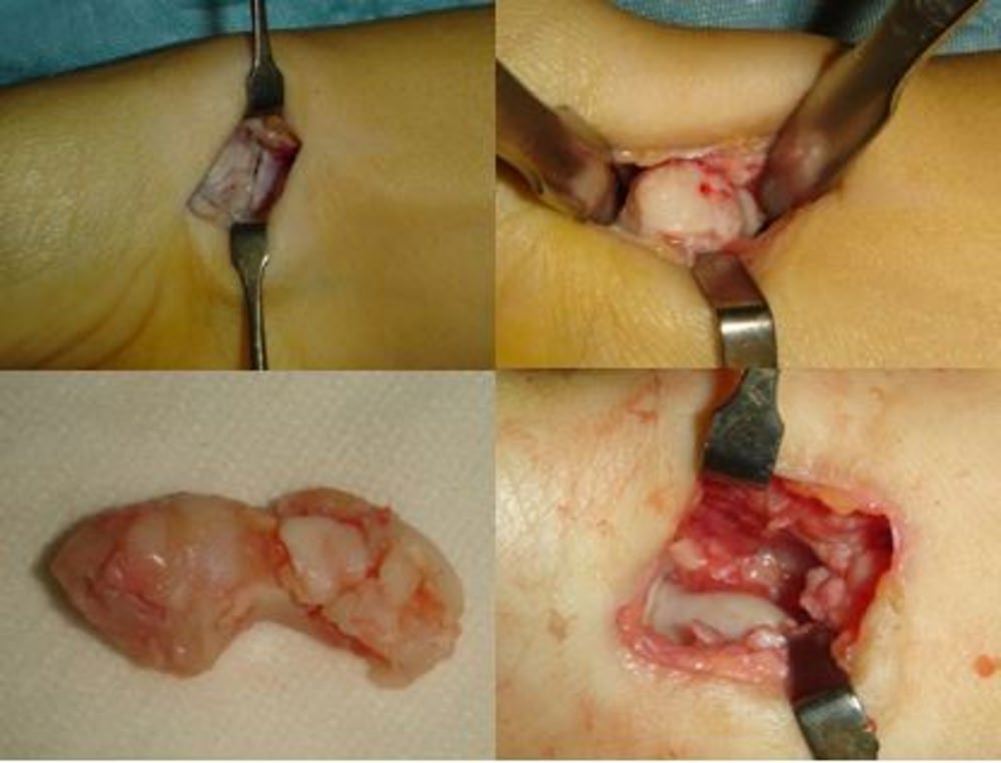
Intra-operative view of the proposed surgical procedure

Cefazolin 2 g was administered intravenously one hour before the procedure in all patients. All the procedures were performed under axillary block anaesthesia, using a volar approach to the scaphoid. The incision was in line with the flexor carpi radialis (FCR) tendon. The superficial palmar branch of the radial artery which passes towards the palm, running close to the scaphoid tubercle, was identified and, if necessary, ligated and divided. The palmar wrist capsule and the radioscaphocapitate ligament were opened longitudinally, and the entire scaphoid was exposed, preserving the palmar ligament complex as much as possible to close properly the capsule after the bone removal. The scaphoid was freed from its ligament attachments and capsule and, then, removed (Fig. 1). A splint was placed for 2 weeks in all the cases. Then, the patient was allowed to progressively recover his wrist range of motion (ROM), followed by strengthening exercises at 4 weeks. Returning to a manual work was recommended no earlier than 8 weeks after surgery.

### Post-operative evaluations

Patients were reviewed at 8-weeks post-op (first evaluation), in 2012 (1 to 6 years follow-up, mean: 4.3 years; second evaluation) and a third time in 2024 (12 to 17 years follow-up, mean: 14.6 years; third evaluation). The mean follow-up of our study was 15 years (min: 12; max: 17; SD: 2.71) (Table 1).

**Table 1:** Demographics of the enrolled patients

Patient	Age	Side	Disease	Sex	Symptoms duration (years)
1	60	Left	SNAC	M	2
2	50	Right	SNAC	F	2,4
3	65	Right	SLAC	M	1
4	55	Right	SNAC	M	6
5	56	Right	SNAC	M	4,3
6	54	Left	SLAC	M	4,1
7	50	Left	SNAC	M	4,8
8	55	Right	SNAC	M	1,2
9	62	Right	SLAC	F	2,3
10	60	Right	SNAC	M	2,4
11	61	Left	SNAC	M	2,9
12	52	Right	SLAC	M	4,8
13	57	Right	SLAC	M	5,1
13*	57	Left	SLAC	M	4,8

Pre-operative clinical data were compared with data obtained during the first evaluation. Then, data from all the three post-operative evaluations were compared between them and with pre-operative data. Clinical evaluation included range of motion (ROM) and grip strength measured by a Jamar dynamometer. Subjective outcome was evaluated by a pain rating score from 1 to 10 (VAS score) and by the Manchester modified DASH score [22, 23].

Radiographic assessment was evaluated in all the 14 wrists at the second evaluation and in 6 at the longer term one (third evaluation). The radiographic evaluation included measurement of the Youm’s index [24] pre-operatively, at the second and third evaluations and was performed blindly by 2 authors and in case of discordance the senior author solved the divergences.

Due to the time elapsed from the surgical procedure in 4 patients was only possible to have a telephone interview: in these cases, only clinical feedback was obtained by asking patients the modified DASH score questions in a telephone interview to avoid potential bias. These patients received a copy of the clinical score and of the informed consent by email days before the interview.

All patients enrolled were invited to sign the informed consent. The study was carried out in accordance with the principles of the Declaration of Helsinki and approved by the local Ethics Committee.

### Statistical analysis

Statistical analysis was performed using SPSS (IBM Statistics) (chi-square, t-test, Mann-Whitney). A statistical confidence level of 95% was selected. A p value < 0.05 determined significance.

## Results

The mean pre-operative VAS score was 5,3 (min: 4,1; max: 9; SD: 2,53). Pain improved during the post-operative time, reaching a mean of 1,53 (min: 0; max: 5,2; SD: 1,55) at the first post-operative evaluation. This improvement was statistically significant (t 5.174 *p* < 0,05).

**Fig. 2:**
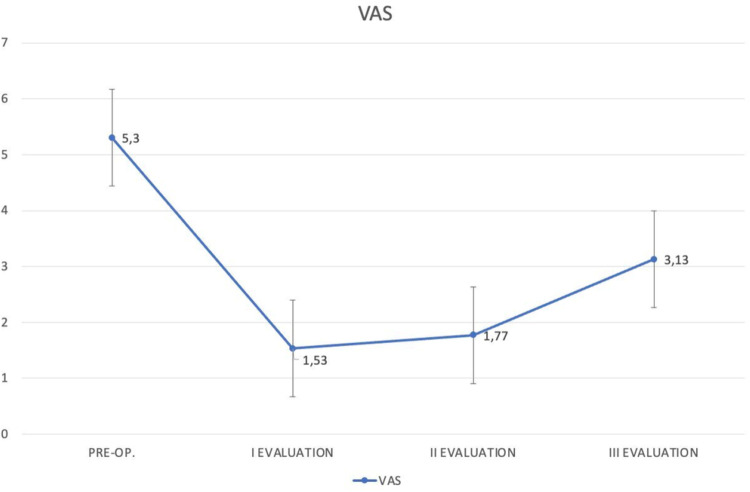
Diagram of the VAS score

Comparing VAS score at the first and second post-operative evaluations, the authors did not find a statistically difference (t -0.403). On the other hand, worsening of pain was recorded at the third evaluation, obtaining a statistically difference between the first and the third one (t -2.176 *p* < 0,05) (Fig. 2).

**Fig. 3:**
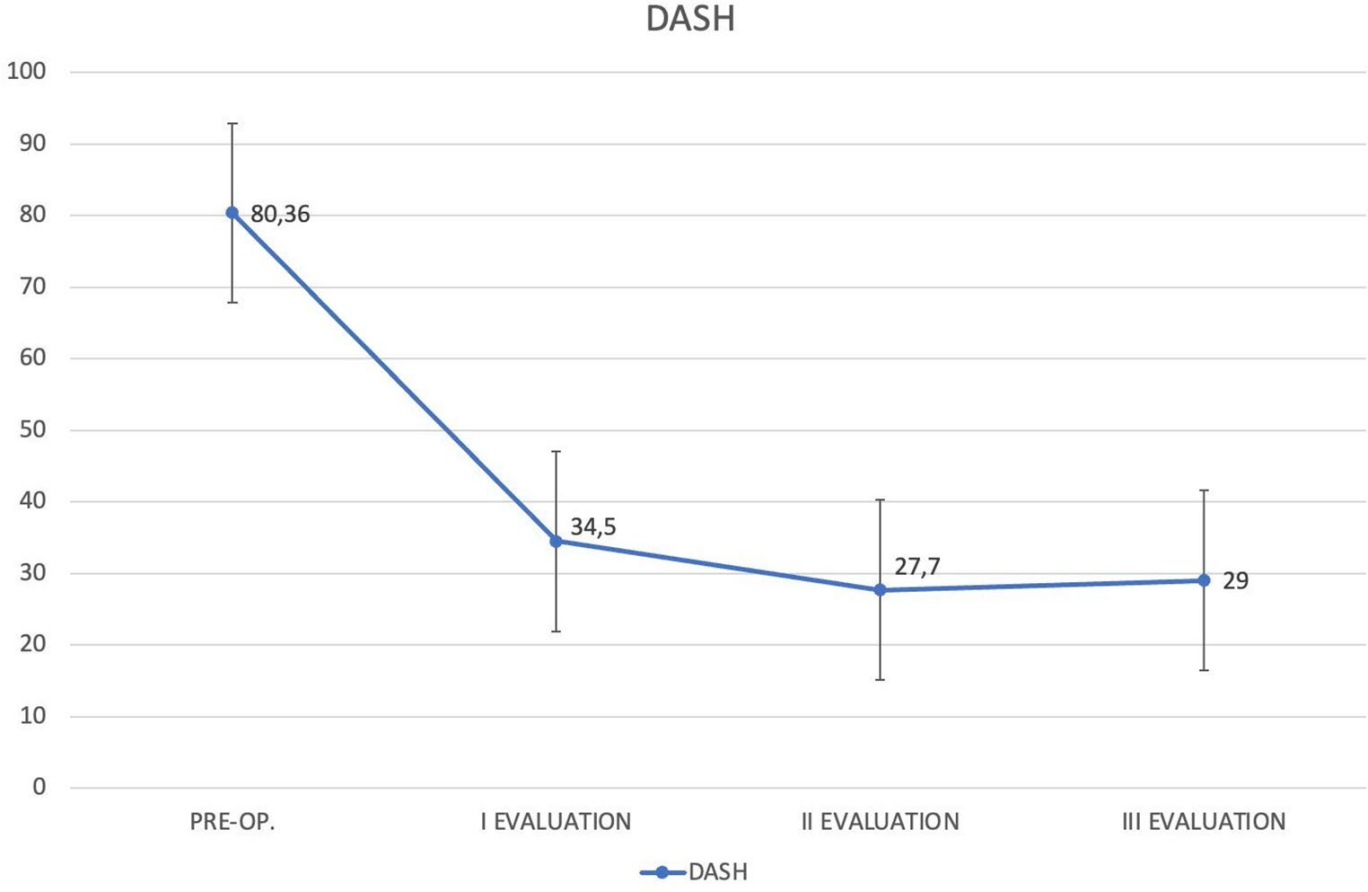
Diagram of the modified DASH score

Similarly, the Manchester modified-DASH score improved significantly from the pre-operative condition to the first evaluation (t 8.481 *p* < 0,05), resulting stable at the second evaluation (t -1.296). The difference does not appear significant when comparing the values recorded at the second and third evaluations (t -1.296) (Fig. 3).

Pre-operative grip strength was significantly lower than the contralateral hand (t -7.249; *p* < 0,05). This difference persisted at the first evaluation (t -3.953; *p* < 0,05) and at the second evaluation and at the third one (t 8,156; *p* < 0,05). But if the pre-operative grip values were compared with those obtained 8 weeks after the surgery, the increase in strength of the affected hand was statistically significant (t -4,853; *p* < 0,05).

**Fig. 4:**
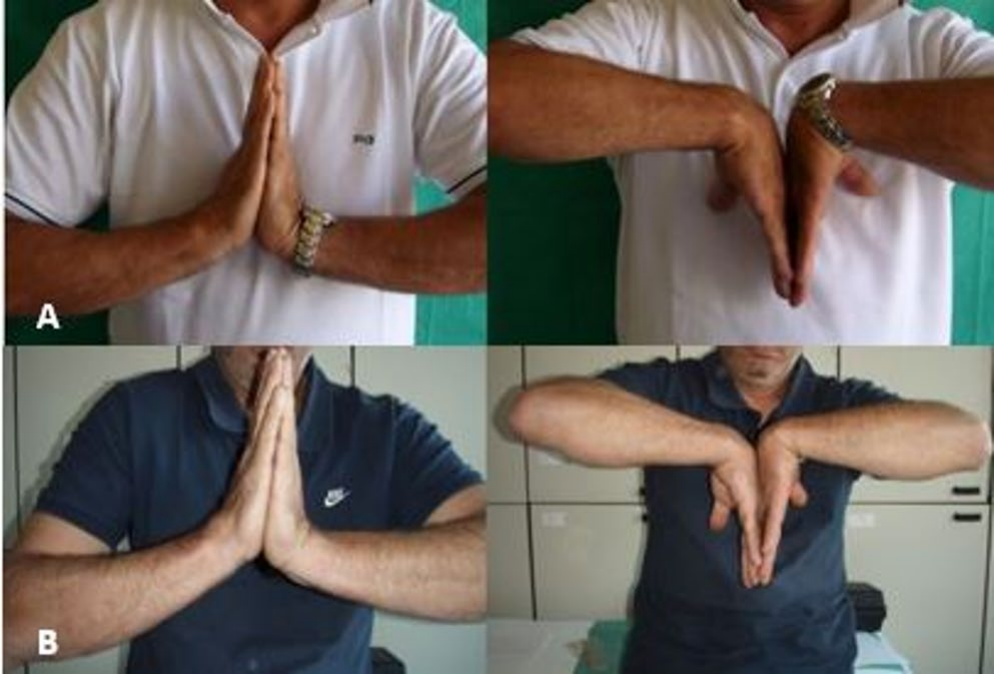
Wrist ROM in the same patient 8 weeks (**A**) and 12 years (**B**) after the procedure

**Fig. 5:**
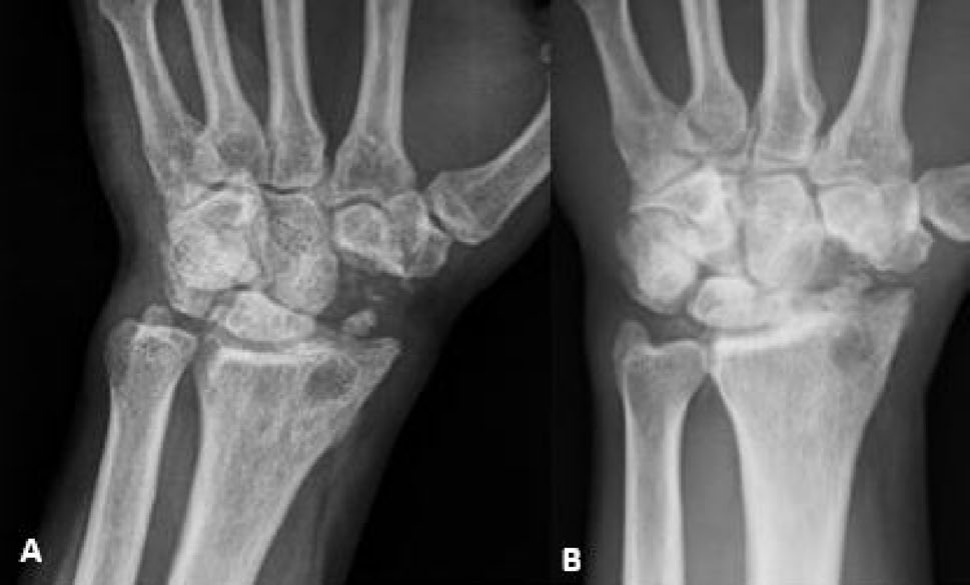
Wrist and hand X-ray in the same patient 8 weeks (**A**) and 12 years (**B**) after the procedure

The mean pre-operative flexion-extension wrist range of motion was 100,8 (min: 95; max:105; SD: 2,77), but the 8- week follow-up range was 91,93 (min: 87; max: 98; SD: 3,24), recording a worsening in the ROM (t 7,757 *p* < 0,05). Similarly, radio-ulnar deviation got worse (pre-op: 33,9; min: 30; max: 37; SD: 2,02–8-week post-op: 25,4; min: 21; max: 30; SD: 5,22 – *p* < 0.05). On the other hand, at the long-term follow-ups, the authors recorded no significative further deterioration of the wrist ROM (Figs. 4 and 5).

Radiographic assessment was done in all the 14 wrists at the second evaluation and in 6 cases at the last long-time evaluation. Radiological deterioration was seen in 6 of the 14 wrists at the second evaluation and in all the 6 patients that could be investigated with an X-ray at the second long term follow-up (Suppl. Mat. 1). Lunate-Capitate arthrosis and a DISI pattern were the radiological features observed but there was a very poor correlation with the clinical outcome. No carpal collapse occurred among the enrolled patients, with Youm’s index maintained in all the patients. Youm’s index averaged 0.55 (min: 0.51; max: 0.57; SD: 0.03) prior to surgery and 0.51 (min: 0.49; max: 0.55; SD: 0.03) at second follow-up and 0.50 (min: 0.48; max: 0.53; SD: 0.02) at the third evaluation, without obtaining a statistically difference between the three different observations while highlighting the small sample size at the last follow-up (t: 2.425).

Only one patient underwent Four Corner Fusion 2 years from the scaphoidectomy for persistent pain. 12 of 13 patients had resumed the same work at a mean of 3 months after surgery. Of the remaining 12 patients, 11 declared their satisfaction for the procedure at the longest follow-up.

We also reported clinical outcomes in comparison of the 2 different surgical techniques used to treat SNAC/SLAC type II (Isolated Scaphoidectomy and Four-Corner Arthrodesis) in 2 homogeneous cohort of patients in Table 2.

**Table 2:** Clinical outcomes at similar follow-up in 2 homogeneous cohort of patients who underwent Isolated Scaphoidectomy and Four-Corner Arthrodesis for SNAC/SLAC type II

	Isolated scaphoidectomy	Scaphoidectomy and 4-C arthrodesis
Number of patients	13	9
Sex (M; F)	11;2	9;0
Age (years)	56.5 (50–65)	57 (51–67)
Follow-up (months)	180 (144–204)	132 (72–156)
Pain score	3.2 (0-4.5)	2.8 (1-4.5)
Grip strenght	28 Kg	32 Kg
Flexion-extension	91.6° (85–97)	88° (75–96)
Radial-ulnar deviation	23° (20–29)	28° (22–32)
Modified DASH score	29 (15–40)	25 (13–46)
Complications	1: four corner fusion after 2 years for persistent pain without collapse	1: pins infection

## Discussion

Many surgical treatment options of symptomatic SNAC and SLAC wrists have been proposed, ranging from radial styloidectomy to total wrist arthrodesis according to the radiological stage. An accurate preoperative imaging study and sometimes a diagnostic arthroscopy, as suggested by Luchetti, are essential to decide which is the best surgical approach [25, 26].

In Stage II the radio-scaphoid joint is involved, while the midcarpal joint is still substantially spared. Four Corner Fusion and Proximal Row Carpectomy are the most recommended options for stage II SLAC and SNAC wrists supported by a wide consensus in the literature. However, less invasive procedures have been proposed over the years to avoid overtreatment and to spare the midcarpal joint still free of disease. Malerich et al. reported their data about a cohort of 19 patients who underwent distal scaphoid resection for SNAC, showing good results in terms of ROM, grip and pain. The authors reviewed clinical and radiographical outcomes also after 15 years of follow-up demonstrating good clinical results despite progressive, but asymptomatic, midcarpal osteoarthritis. Only 2 patients underwent proximal row carpectomy and wrist arthrodesis for recalcitrant pain [18].

The major issue associated with isolated scaphoidectomy is the risk of midcarpal collapse and dorsal intercalated segment instability (DISI). It is known that scaphoidectomy leads to dorsal extension of the lunate and DISI pattern, but this biomechanic and radiographical feature is often asymptomatic. DISI does not always correlates with bad clinical scores. Brouwers et al. studied the DISI incidence after distal scaphoidectomy associated with tendon interposition (FCR) in 18 patients and found that 95% of the enrolled patients showed radiographic signs of DISI at the short-term follow-up, but there were no cases of functional impairment or increased pain [27].

In 2003, Soejima et al. published their results after distal scaphoid resection in case of SNAC in 9 patients, mostly males, with a follow-up of 29 months. Confirming how effective is this treatment, patients performed good, obtaining excellent clinical scores and no progression of the osteoarthritis on the X-rays in 8 patients [17].

To optimize the surgical procedure, other techniques have been proposed over time to postpone more complex and invasive ones. For example, scaphoidectomy associated with a midcarpal tenodesis has been proposed by Luchetti in young patients to avoid more invasive techniques in Stage II disease [20, 26].

In 2012, Trumble et al. proposed to treat SNAC/SLAC stage II with scaphoidectomy associated with capsulodesis through a dorsal surgical approach. The study included 8 patients with a short-term follow-up of 16 months with a mean age of 62 years. They obtained a good wrist ROM and grip strength with improved pain scores. They also found some midcarpal osteoarthritis deterioration but with a poor correlation to the clinical outcome.

**Table 3:** Comparison of data reported in our study with those of Trumble et al. [21]

	Our study	Trumble et al. [21]
Number of patients	13	8
Sex (M; F)	11; 2	7;1
Age (years)	56.5 (50–65)	62
Follow-up (months)	180 (144–204)	24 (20–30)
Surgical approach	Volar Approach	Dorsal Approach
Pain score pre-op.	5.3 (4.1-9)	8.5 (6–10)
Pain score post-op.	1.5 (0-5.2)	2.4 (1–5)
Grip strenght pre-op.	19.2 Kg	18 Kg
Grip strenght post-op.	28 Kg	28 Kg
Flexion-extension pre-op.	100° (95–105)	110°
Flexion-extension post-op.	92° (87–98)	100°
Radial-ulnar deviation pre-op.	34° (30–37)	40°
Radial-ulnar deviation post-op.	25.4° (21–30)	38°
DASH score pre-op.	80 (70–100)	Not reported
DASH score post-op.	34.5 (10–61)	21 (16–38)
Youm’s index pre-op.	0.55 (0.51–0.57)	0.48
Youm’s index at the II F.U.	0.51 (0.49–0.55)	0.46
Failures	1: four corner fusion after 2 years for persistent pain without collapse	Not reported

Our reported clinical outcomes are at the fist post-operative evaluation to make the two groups comparable due to the difference in the follow-up duration

As reported in our cohort of patients, the wrist ROM decreased in the post-operative time but with good pain control and satisfactory clinical scores [21].

We treated 14 wrists affected by SNAC/SLAC Stage II with an isolated scaphoidectomy performed by a palmar approach. This surgical option was offered to type II SLAC and SNAC wrists independently from age, gender and work. Based on the encouraging outcome of distal scaphoid resections [16–18] and data from Trumble et al. [21], in case of type II SNAC, we hypothesized that an isolated scaphoidectomy could provide a satisfactory pain release and could be supplemented by the 4CF or proximal row carpectomy only at a later time, if needed.

Patients first review at a mid-term follow-up (1–6 years) was encouraging. All patients resumed their previous work with good pain and strength scores. Only one patient underwent a 4CF two years after the scaphoidectomy. Radiological deterioration was seen in 6/14 wrists and a DISI pattern of instability and mild midcarpal osteoarthritis were the major radiological features observed, as expected. However, these radiological findings did not correlate with pain and clinical outcomes.

Our radiographic results are in line with that reported by Trumble [21] and Soejima [17] but in contrast with studies reporting a high rate of carpal collapse following SNAC/SLAC [28–30].

The long-term review with a follow-up ranging from 12 to 17 years has confirmed that the isolated scaphoidectomy maintains a high level of satisfaction over the years despite a progressive radiological osteoarthritis deterioration.

We also compared our data with those reported by Trumble in Table 3.

## Conclusions

Four Corner Fusion and Proximal Row Carpectomy are well established treatment for stage II SLAC and SNAC wrists supported by high consensus in the literature. Based on the long-term survey of a small group of patients, authors suggest that isolated scaphoidectomy by volar approach can be a surgical option. It is far less invasive and simple to perform and requires a shorter time of recovery. This procedure has maintained a high level of satisfaction at 12 to 17 years of follow-up despite the radiological osteoarthritis deterioration. In case of failure, more invasive techniques can be performed at any time.

Isolated scaphoidectomy should be considered a reasonable and simple surgical procedure to be reserved for elderly patients or ones with low functional demands. The aim of this surgical procedure is primarily pain control. Prospective studies should be useful to better define the role of this surgical option in type II SNAC/SLAC wrists.

Our study has some limitations: this is a retrospective study with a small sample size, and some patients were lost at the last follow-up or reachable only by telephone (no radiological data available). On the other hand, the follow-up is the longest one in the literature about this topic, and the cohort of the enrolled patients is homogenous for demographic characteristics.

The aim of the study is to demonstrate that the technique is reliable and less invasive than other proposed alternatives, which certainly pose a lower risk of carpal collapse, although the latter was not observed in the cohort of patients analysed. Absence of the scaphoid results in a pattern of instability with radiographic evidence of DISI, but with good pain control and wrist range of motion. Our patients have not experienced worsening symptoms despite physiological joint aging of the carpal bones (lunate-capitate osteoarthritis) and DISI, but without actual carpal collapse with significant worsening of the Youm’s index. Since there are few studies on the isolated scaphoidectomy and with such a long follow-up, it is difficult to compare our radiological results with others. However, a recent study investigating how DISI following distal scaphoid resection and tendon interposition affects hand function shows that it does not correlate with functional scores and these outcomes are in line with the data found in our case series [27].

Therefore, careful selection of patients undergoing this technique is mandatory. Patients should be low function demanding, aged over 50 years, with type II SNAC or SLAC wrists. These patients need pain relief but do not want to undergo more extensive surgical procedures or refuse prolonged immobilization as requested in case of arthrodesis.

## Supplementary Information

Below is the link to the electronic supplementary material.

Supplementary Material 1 Hand and wrist X-rays of the 6 enrolled patients at the longest follow-up

## Data Availability

The data presented in this study are available on request from thecorresponding author.
