# Supplementary material for: Isolated scaphoidectomy for type II SLAC and SNAC wrists: retrospective case-series at long-term follow-up
Source: Arch Orthop Trauma Surg. 2026 May 24;146(1):196. doi: 10.1007/s00402-026-06350-z (PMC13199185; doi:10.1007/s00402-026-06350-z)
Supplement: Supplementary file 1 — Supplementary Material 1 Hand and wrist X-rays of the 6 enrolled patients at the longest follow-up [file 402_2026_6350_MOESM1_ESM.pdf]

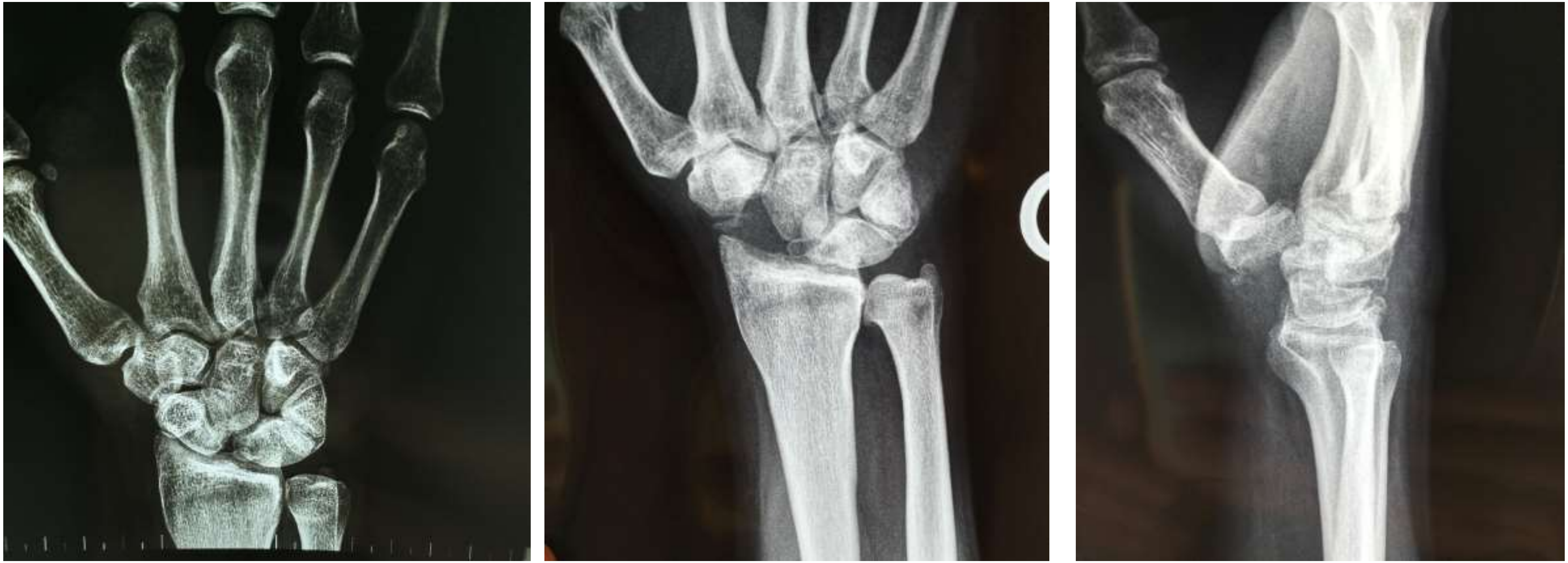

F.U.: 12,3 years
Youm’s Index: 0.49
Vas: 1.5
DASH: 29
Grip: 28 Kg
Flex-Ext: 91°
Radial-Ulnar deviation: 24°

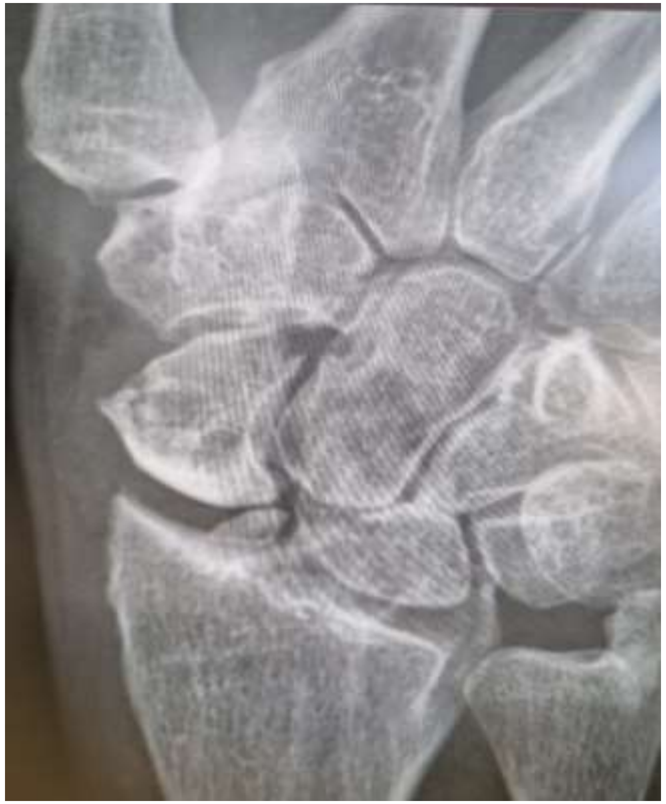

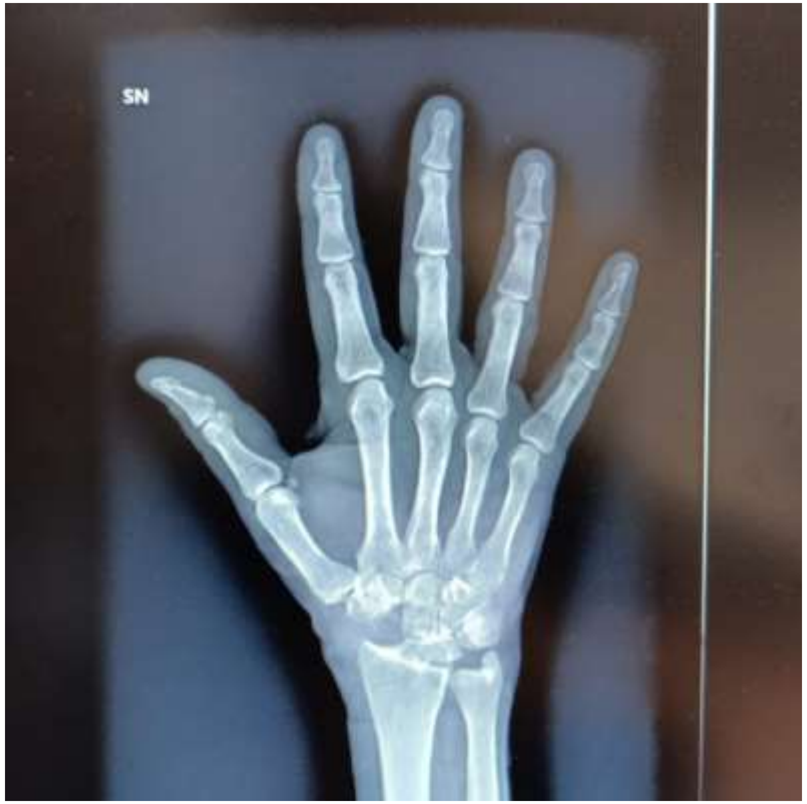

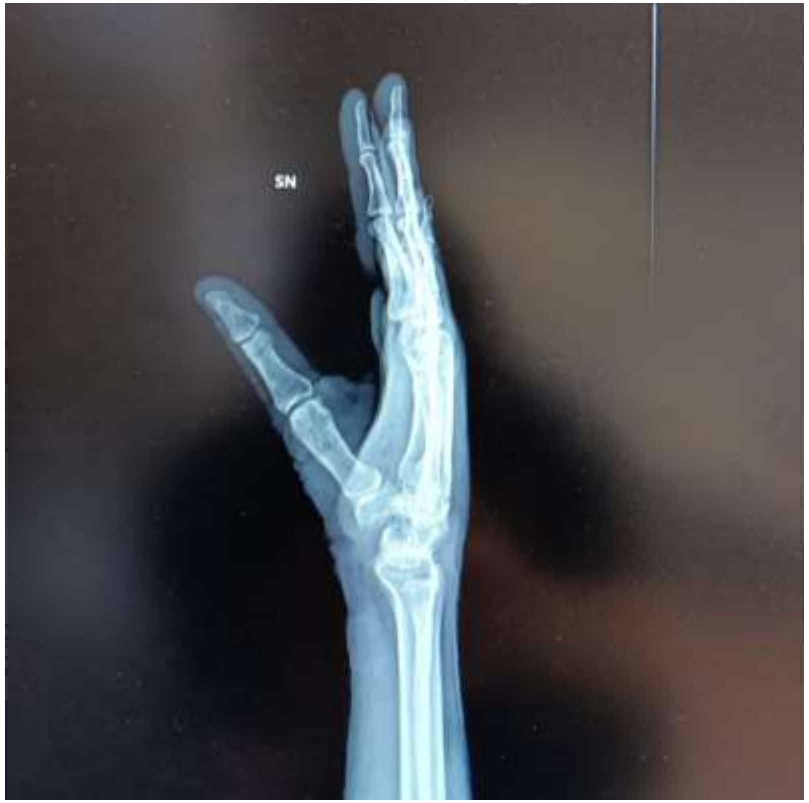

F.U.: 13,4 years
Youm’s Index: 0.50
Vas: 3
DASH: 30
Grip: 28 Kg
Flex-Ext: 97°
Radial-Ulnar deviation: 29°

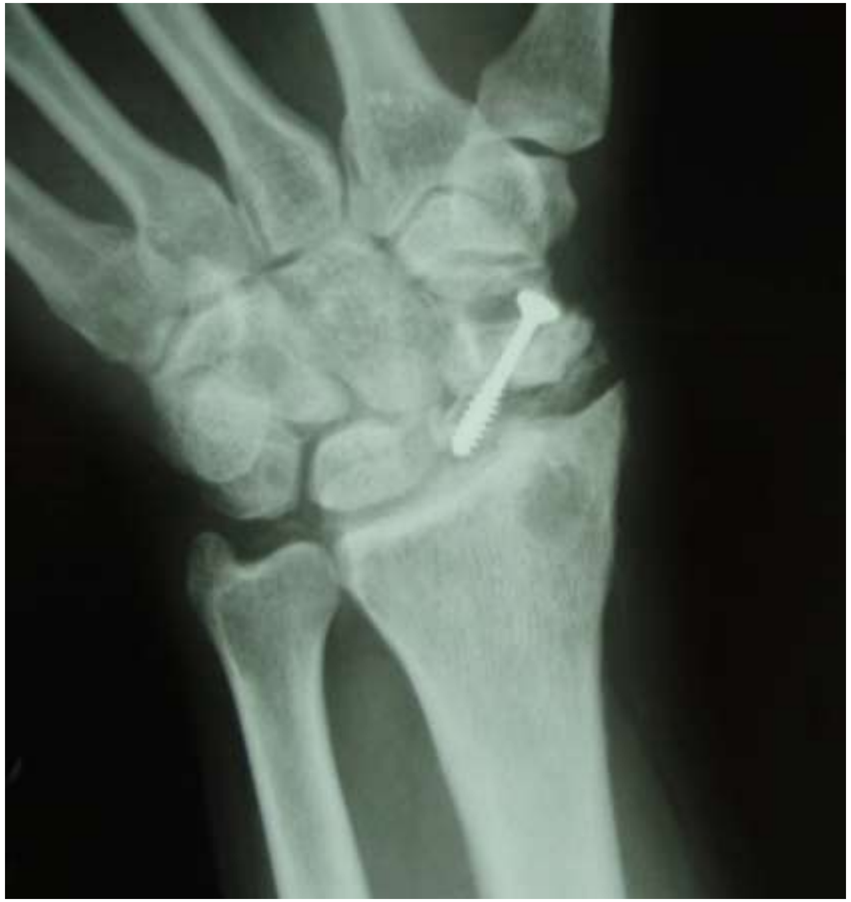

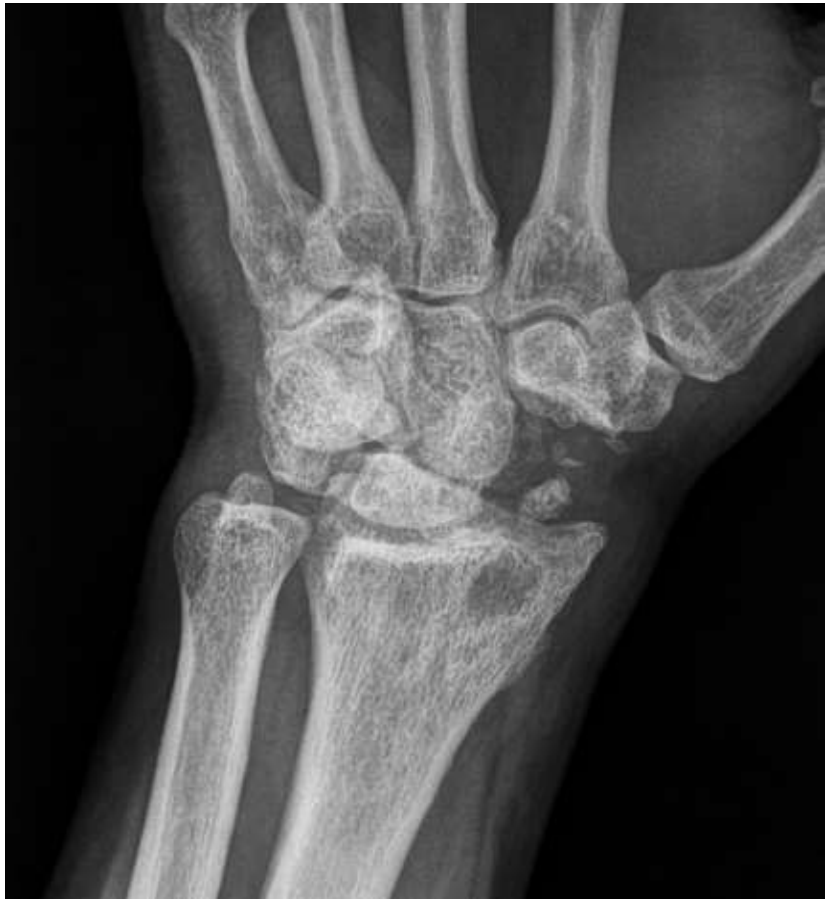

F.U.: 15 years
Youm's Index: 0.52
Vas: 4
DASH: 30
Grip: 29 Kg
Flex-Ext: 91°
Radial-Ulnar deviation: 20°

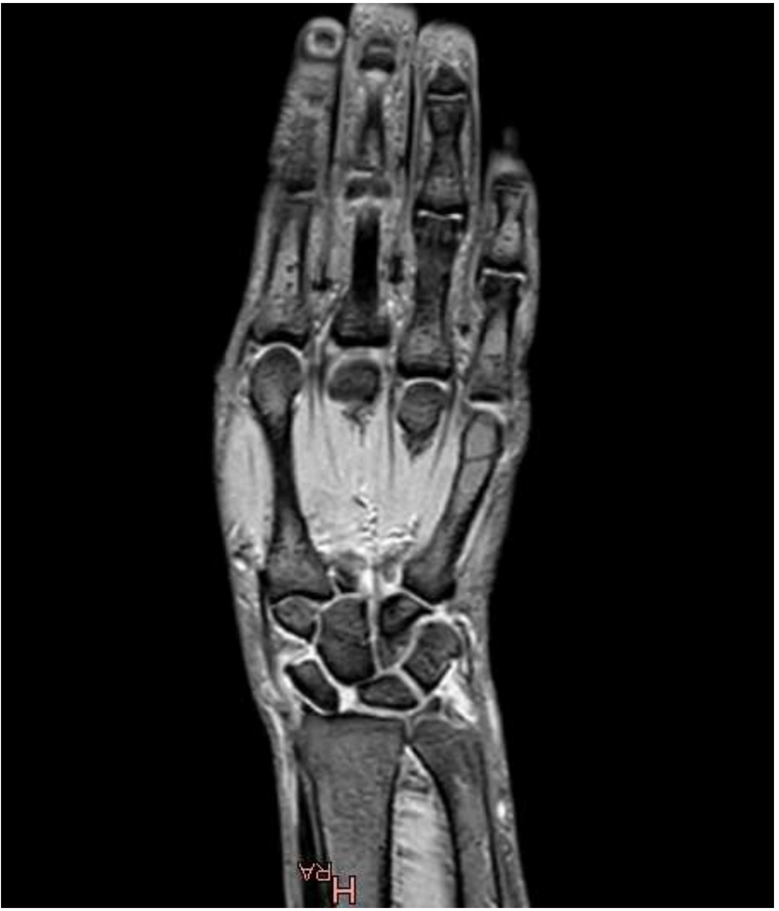

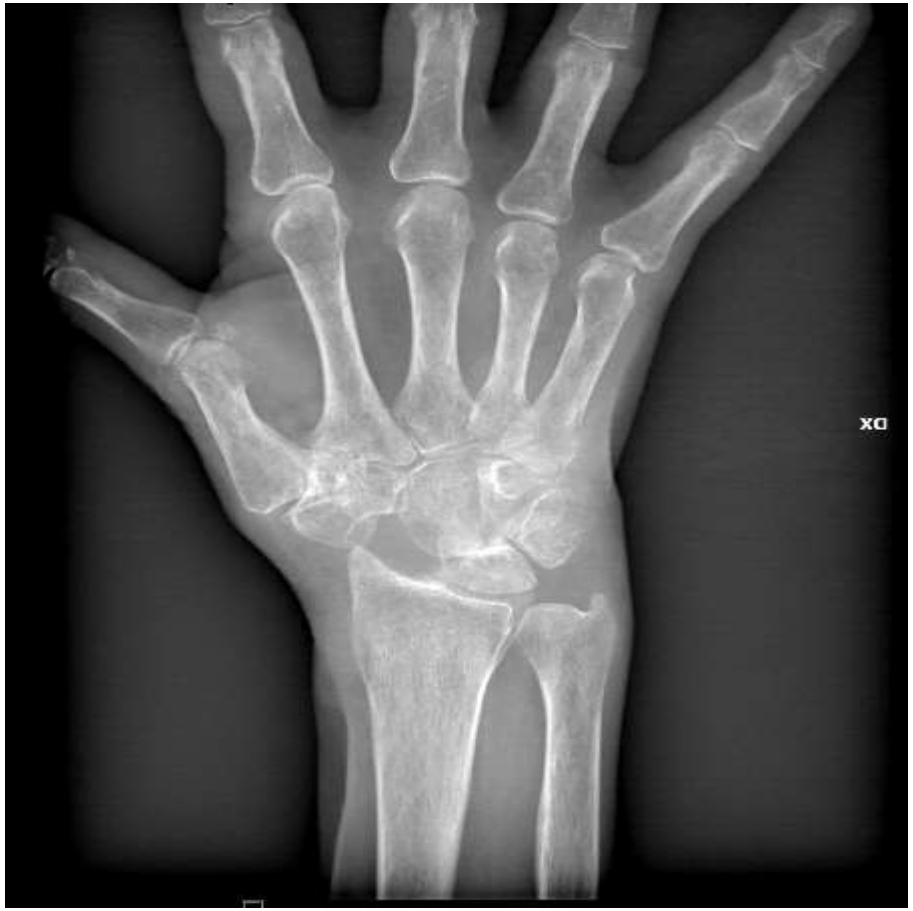

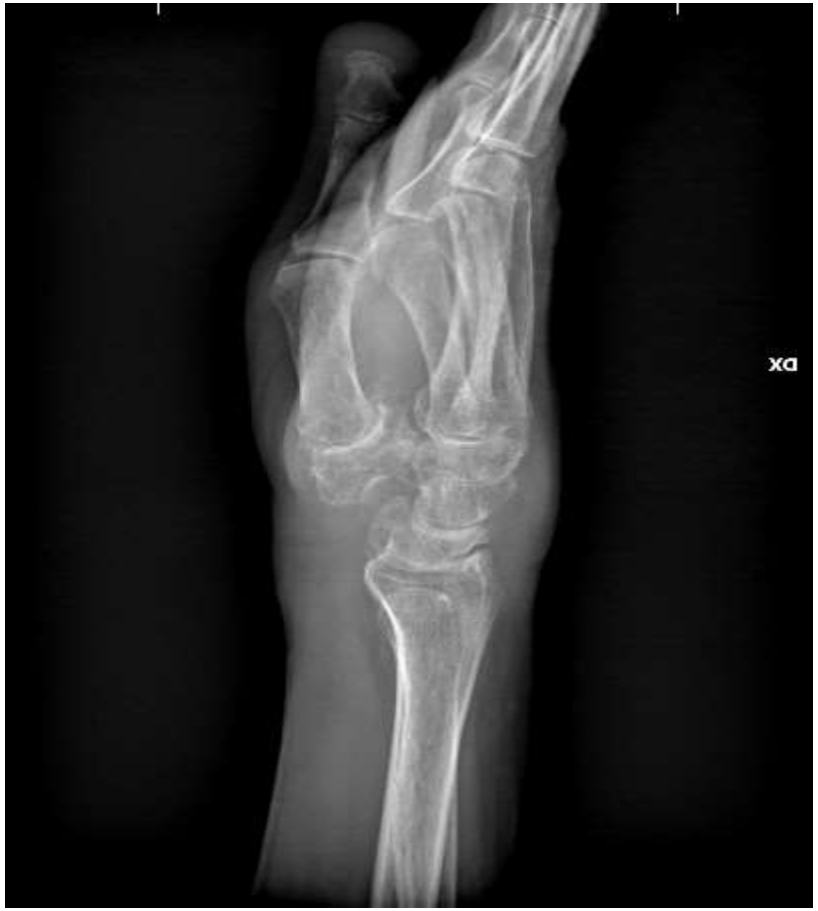

F.U.: 17 years
Youm's Index: 0.48
Vas: 4.5
DASH: 40
Grip: 25 Kg
Flex-Ext: 85°
Radial-Ulnar deviation: 20°

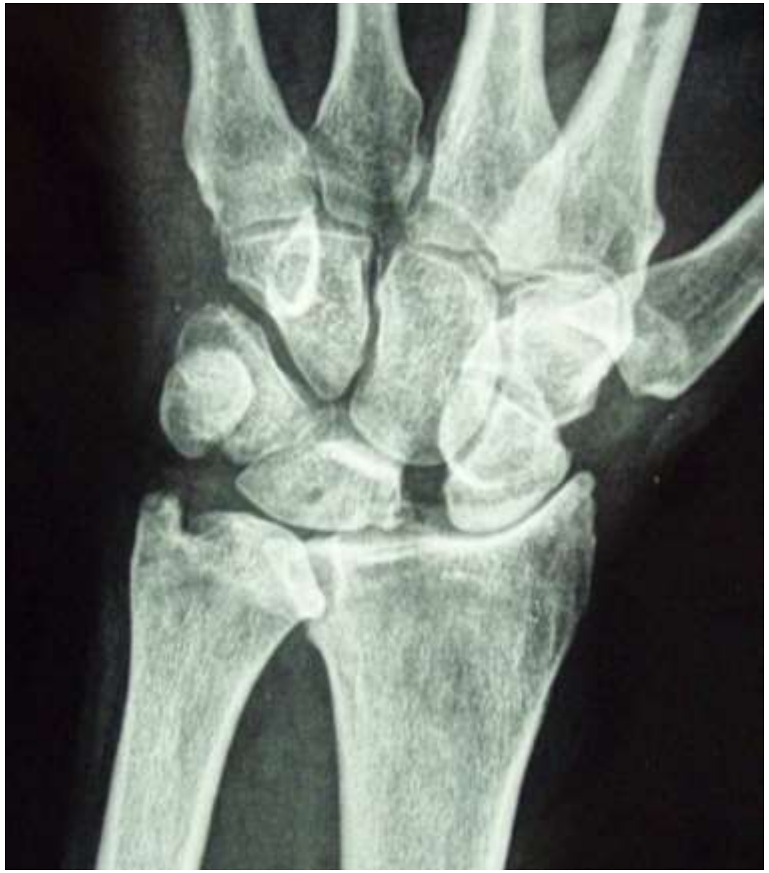

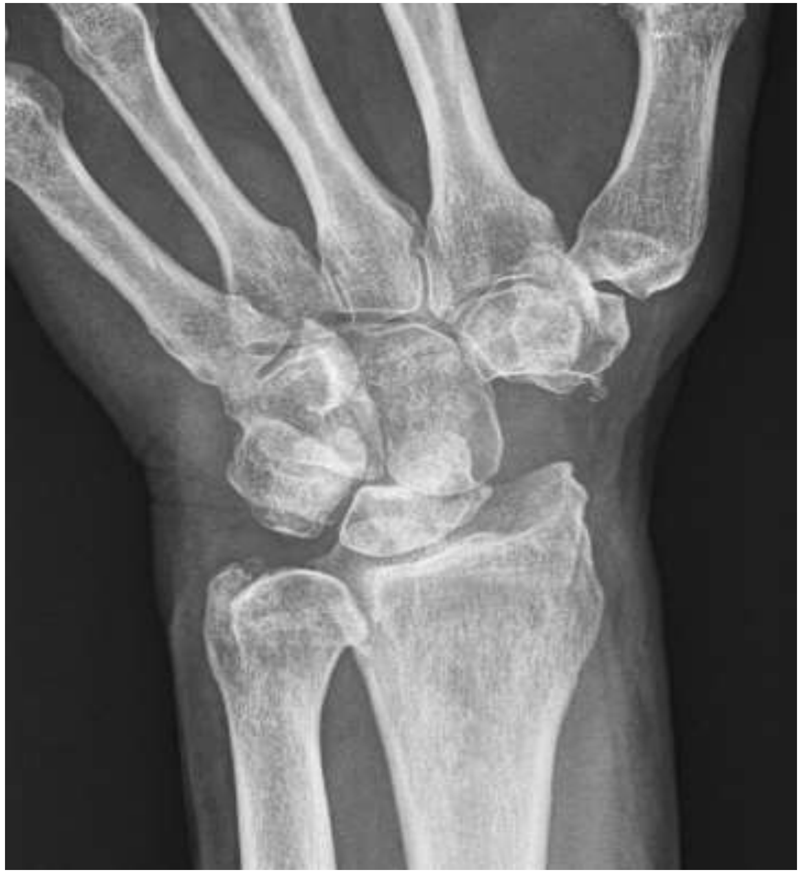

F.U.: 14.5 years
Youm’s Index: 0.53
Vas: 3
DASH: 15
Grip: 30 Kg
Flex-Ext: 94°
Radial-Ulnar deviation: 24°

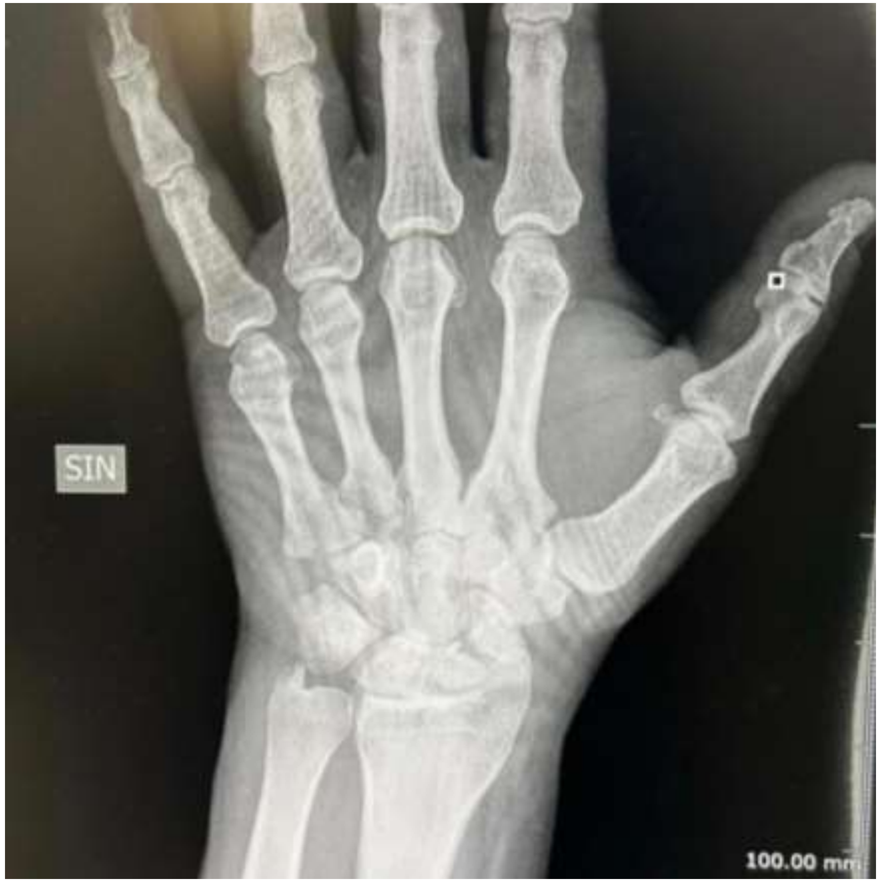

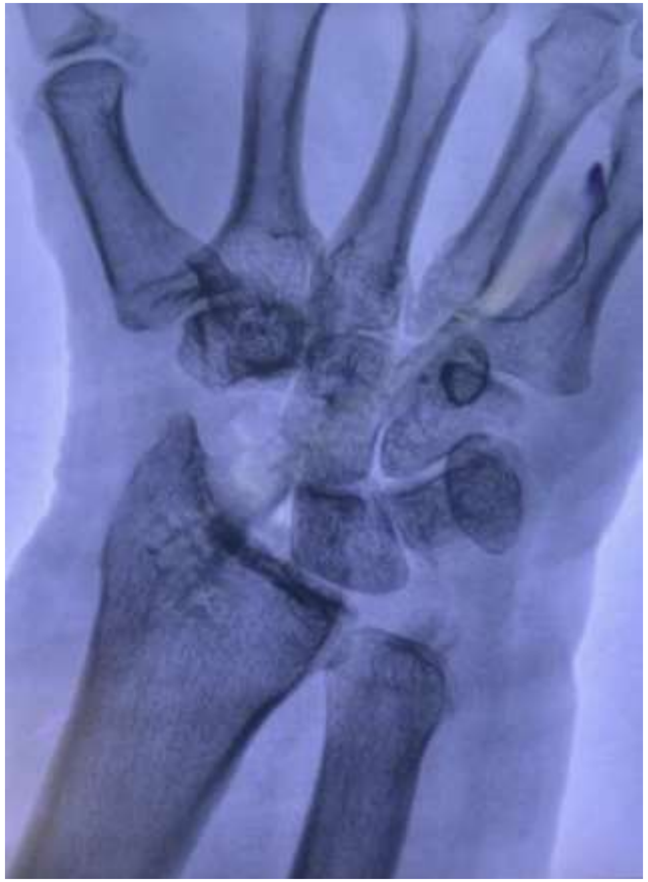
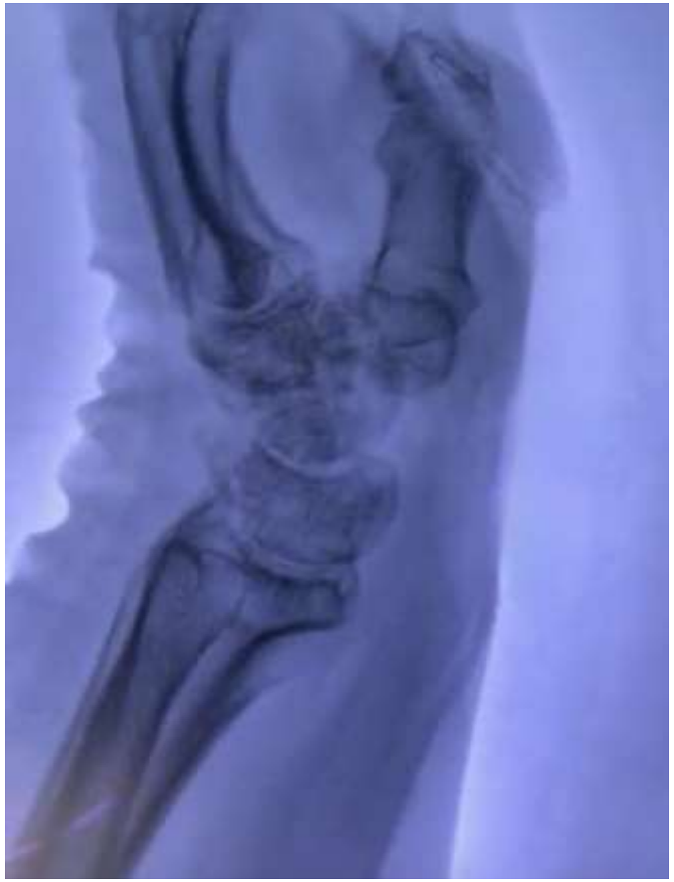

F.U.: 15.3 years
Youm's Index: 0.50
Vas: 3
DASH: 29
Grip: 25 Kg
Flex-Ext: 85°
Radial-Ulnar deviation: 20°
